# Large-Scale Analysis of Network Bistability for Human Cancers

**DOI:** 10.1371/journal.pcbi.1000851

**Published:** 2010-07-08

**Authors:** Tetsuya Shiraishi, Shinako Matsuyama, Hiroaki Kitano

**Affiliations:** 1 Sony Computer Science Laboratories, Shinagawa-ku, Tokyo, Japan; 2 Sony Corporation, Shinagawa-ku, Tokyo, Japan; 3 The Systems Biology Institute, Shinjuku-ku, Tokyo, Japan; 4 Okinawa Institute of Science and Technology, Kunigami, Okinawa, Japan; University of Illinois at Urbana-Champaign, United States of America

## Abstract

Protein–protein interaction and gene regulatory networks are likely to be locked in a state corresponding to a disease by the behavior of one or more bistable circuits exhibiting switch-like behavior. Sets of genes could be over-expressed or repressed when anomalies due to disease appear, and the circuits responsible for this over- or under-expression might persist for as long as the disease state continues. This paper shows how a large-scale analysis of network bistability for various human cancers can identify genes that can potentially serve as drug targets or diagnosis biomarkers.

## Author Summary

Since most disease states exhibit a certain level of resilience against therapeutic interventions, each disease state can be considered to be homeostatic to some extent. There must be one or more mechanisms that cause the gene-regulatory network to maintain a certain state, and one such mechanism is a bistable switch. In this work, bistable switch networks were constructed and their ON(upregulated)/OFF(downregulated) states were compared between human cancers and healthy control samples. Changes in the ON/OFF state with the progression of cancer were demonstrated. A series of genes that might serve as a drug target or diagnosis biomarker was identified. The approach presented here should provide useful insights into the states of biological networks, which may lead to the discovery of novel drug targets and therapeutic interventions.

## Introduction

**Figure 1:**
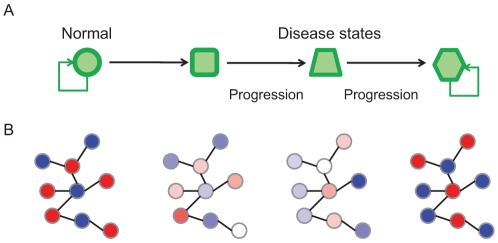
State transitions in network dynamics and disease progression. A: A network in a healthy state is robust against a range of perturbations, so it can continue to maintain a healthy state. With the onset of a disease, however, the network transitions to a new steady state that is also often robust against perturbations, making the disease state persistent. B: These state transitions might be driven by bistable switch networks. The nodes represent genes and the edges between them represent the pairing of bistable toggle switches. Red and blue nodes correspond to ON (upregulated) and OFF (downregulated) states.

Understanding diseases within the context of biological networks is one of the major challenges in systems biology. Diseases often persist and resist therapeutic intervention. The persistence of a disease in a system must be reflected in the ability of the system's networks to maintain the state underlying the disease. In other words, networks are “locked-in” to disease states and maintain their stability. Thus, it is important to understand how such multi-stable states are achieved within the context of network topology and to understand the dynamics of these states. A network robust against a range of perturbations can maintain a healthy state but can also, when affected by a disease, transition to a new steady state that is often also robust against perturbations, making the disease state persistent. A series of disease progressions may be the result of a sequence of state transitions in the network dynamics (Fig. 1A). Bistable circuits may drive such transitions and are thus critical in enabling the initiation and progression of diseases to be understood (Fig. 1 B).

Complex networks exhibiting such multi-stability must have a set of bi-stable or multi-stable circuits consisting of proteins and genes. The identification of circuits that exhibit bi- or multi-stability within large protein-interaction and gene-regulation networks would provide information useful for understanding the mechanism(s) of network bistability. Furthermore, circuits exhibiting bistability can be potential drug targets or biomarkers for classifying disease states.

Network dynamics are regulated by the structure of the network and the flow of information through feedforward and feedback loops. Mutual activation or mutual inhibition configurations can maintain the flow of biological information between two molecules and act as network memories or switches. Furthermore, an activation-inhibition configuration, in which one molecule stimulates the other while the latter inhibits the former, generates dynamics with periodicity like that seen in circadian rhythms and cell cycles [1]. The stability and characteristics of Boolean networks comprising these configurations were studied in detail by Kauffman et al. [2]. In the study reported here, we focused on mutual inhibition, which is thought to be involved in the stable deviations of a system observed during the progression of tissue from a normal to a diseased state.

There are several important network motifs for system configurations [3]–[6] in protein-protein networks. One of them, a toggle switch that converts a continuous input signal into a discontinuous ON or OFF response, plays a fundamental role in information processing and decision making. Among the naturally occurring toggle switches that have been reported are the lambda phage lysis–lysogeny switch [7]–[9], switches in the lactose operon repressor system [10]–[12], the mitogen-activated protein kinase (MAPK) cascade [13]–[20], the Sonic hedgehog network in stem-cell differentiation [21], cell-cycle regulatory circuits [22]–[24], and the rapid lateral propagation of receptor tyrosine kinase activation [25]. Genetically engineered toggle switches have been constructed experimentally in Escherichia coli [26], [27] and in mammalian cells [28].

A robust toggle switch behaves as a signal memory unit by using a hysteresis mechanism [29]. Once in the ON state, a toggle switch remains in the ON state even if the stimulus concentration falls below the threshold level [11], [13], [23], [24], [30], [31]. A molecular network's persistence in a disease state might be due to the hysteresis of toggle switches.

To identify circuits exhibiting bi- and multi-stability, we topologically analyzed activation and inhibition in proteins on a large scale by using various databases containing expression array data for various diseases. We compared the progression stages of these diseases with those of control samples by using data for healthy individuals taken from available databases, and we identified sets of switch circuits possibly responsible for maintaining the persistent disease states by using network topologies to analyze that data.

## Results

### Extraction of bistable toggle switches

**Figure 2:**
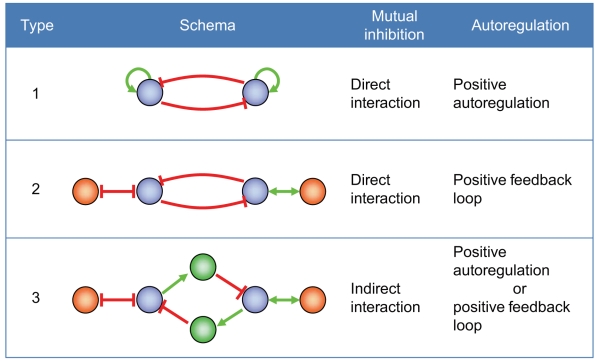
Motifs of bistable toggle switches. A type-1 bistable toggle switch (BTS) contains two genes with positive autoregulation. Each gene mutually inhibits the other's expression. The two genes in the type-2 BTS also suppress each other's expression. Each gene has double positive or negative feedback with the other gene, so the same function as a type-1 BTS may be exhibited. A type-3 BTS was constructed on the basis of a theoretical study on the modeling of genetic switches with positive feedback loops. The blue, green, and orange nodes respectively correspond to switch genes, mediators, and genes constituting a feedback loop. Positive (upregulated) interactions are indicated by green lines and negative (downregulated) interactions are indicated by red lines.

There are theoretically many system configurations that can lead to bistability [18], [32]–[35]. We focused on bistable toggle switches (BTSs) with double-negative feedback. Such switches can be constructed from any two genes that mutually repress their expression. We considered three types of network motifs that can exhibit bistable behavior (Fig. 2).

1. Type-1 BTS: A type-1 BTS uses a basic motif that has been identified in E. coli [26] and has mutually inhibitory interaction and positive autoregulators. In a circuit with a double-negative feedback loop, proteins A and protein B inhibit or repress each other. Positive autoregulation is a type of feedback in which proteins directly activate the transcription of their own genes. Under the right circumstances, there could be a stable steady state in which A is “ON” and B is “OFF” or B is “ON” and A is “OFF.” This bistability is maintained through positive autoregulation.
2. Type-2 BTS: Only a small number of transcription factors with a positive autoregulation ability have been reported. From the viewpoint of dynamic properties, positive autoregulation has the same functional meaning that a positive feedback loop (double-positive feedback or double-negative feedback) does [36]. We thus defined two mutually inhibitory nodes with a positive feedback loop between them as a type-2 BTS.
3. Type-3 BTS: A theoretical study of modeling genetic switches with positive feedback loops [37] revealed that mutual inhibition is maintained even if a molecule that signals information intervenes between the molecules constituting a switch. We defined two nodes that inhibit each other through other genes (mediators) as a type-3 BTS. Although it is theoretically possible that a positive feedback loop can be formed even if the intervening molecules are identical, in the present study we excluded this possibility.

It is possible that double-negative feedback can be a bistable toggle switch when both nodes have positive feedback loops. Two BTSs can share their mutual inhibition configurations as positive feedback loops and can form network configurations.

**Figure 3:**
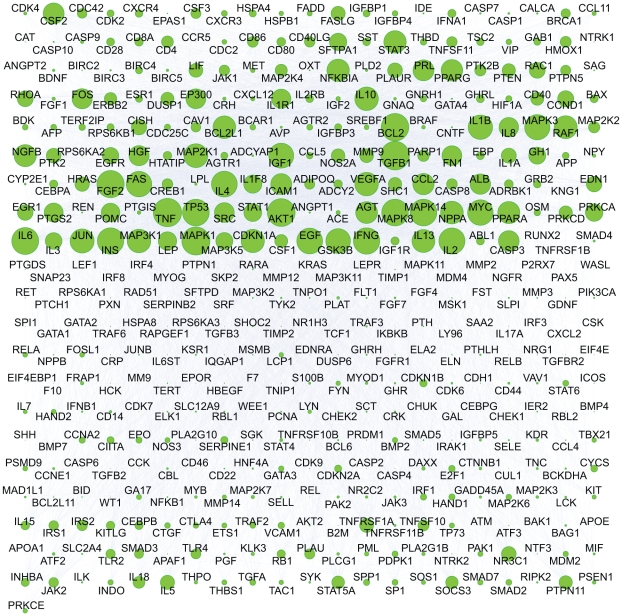
Cytoscape visualization of network composed of bistable toggle switch pairs. Four-hundred and forty-two genes are involved in 6585 bistable toggle switch pairs. Nodes are shown in sizes proportional to their connectivity, making the hubs of switch nodes clearly visible. The Cytoscape session file for this network is available in Protocol S2.

Next, bistable toggle switches defined above was extracted from large-scale databases (ResNet 3.0, Ariadne Genomics Inc.) containing data for interaction networks. We detected 6585 pairs of bistable toggle switches, and these switch nodes formed a large network. Four-hundred and forty-two genes are involved in these BTS pairs, and the hubs of switch nodes in the network are clearly visible because of their high degree of connectivity (Fig. 3). A complete list of the BTS pairs is provided in Protocol S1, and a Cytoscape session file is provided in Protocol S2. It should be noted that this network was constructed using text mining and that the molecular details of each interaction were not verified. It is nevertheless a reasonable starting point, and whether or not a listed BTS actually exhibits bistability can be further examined using microarray data.

### Tests using mRNA microarray data

ArrayExpress microarray data were used to further examine the states of the BTS pairs. It is obvious that a BTS has four possible states: “ON/ON,” “ON/OFF,” “OFF/ON,” and “OFF/OFF.” Mathematical analysis of bistability for the chosen parameter condition demonstrated that the probability of “ON/OFF” and “OFF/ON” states is high, that of “ON/ON” is low, and that of “OFF/OFF” is extremely low [38]. This is the reason we focused on the BTSs that demonstrated “ON/OFF” or “OFF/ON” states.

**Figure 4:**
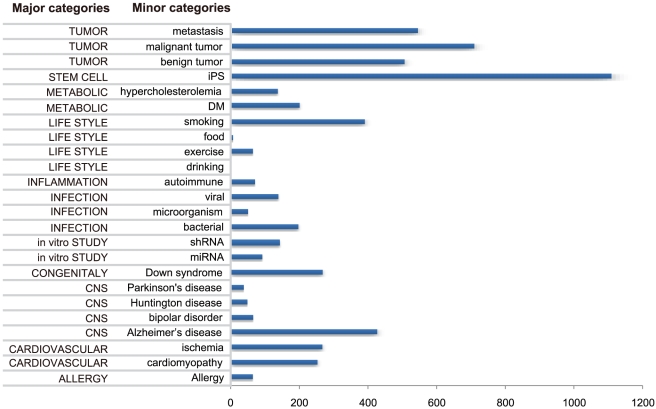
ArrayExpress experimental categories for microarray datasets and mean number of BTS pairs with significant ON/OFF change. There were few BTS pairs with significant changes for “lifestyle” and many with significant changes for “cancer.” Note the higher number of BTS pairs for iPS cells than for donor cells.

The ArrayExpress experimental categories and the mean number of corresponding BTS pairs with a significant ON/OFF change are shown in Fig. 4. In the set of 6585 candidate BTSs the number of pairs with a significant ON/OFF change ranged from 0 to 1927 (mean = 298.6), while in a set of 6585 randomly selected gene pairs the number of pairs with a significant ON/OFF change ranged from 0 to 273 (mean = 72.1).

The switching of a molecule's function to the ON state generally means the molecule's intrinsic function related to intracellular molecular systems has become stronger, whereas switching to the OFF state means it has become weaker. The ON state of a molecule is produced not only by an increase in the absolute amount of that molecule but also by actions such as activation due to phosphorylation-induced transformation of the molecule's three-dimensional structure or to translocation of the molecule to an location where it can carry out its function properly.

In these studies using mRNA expression data from microarrays, the toggling of a BTS pair was defined as an instance in which a sample's mRNA level for one of that pair's molecules increased (relative to a control) and the mRNA level for the other of that pair's molecule decreased (relative to the same control).

A notable finding is that when mRNA levels were compared between induced pluripotent stem (iPS) cells and donor controls, more than 1000 BTS pairs demonstrated significant changes in the ON/OFF states. The high frequency of these changes in iPS cells is reasonable in that an iPS cell is in an undifferentiated state committed to differentiation to a particular lineage, in which many BTSs might be involved [39]. iPS cells have been generated from mouse and human somatic cells by using retroviruses or lentiviruses to introduce Oct3/4 and Sox2 with either Klf4 and c-Myc or Nanog and Lin28 [40]. These factors have been reported to result in bistability when they combine with other factors and form mutual-activation and mutual-inhibition motifs [41]–[43].

### Lung cancer

Lung cancer is the leading cause of cancer-related deaths [44], and tobacco smoking is the strongest etiological factor associated with lung cancer. Prior studies have demonstrated that smoking creates a field of molecular injury throughout the airway epithelium exposed to cigarette smoke [45].

**Figure 5:**
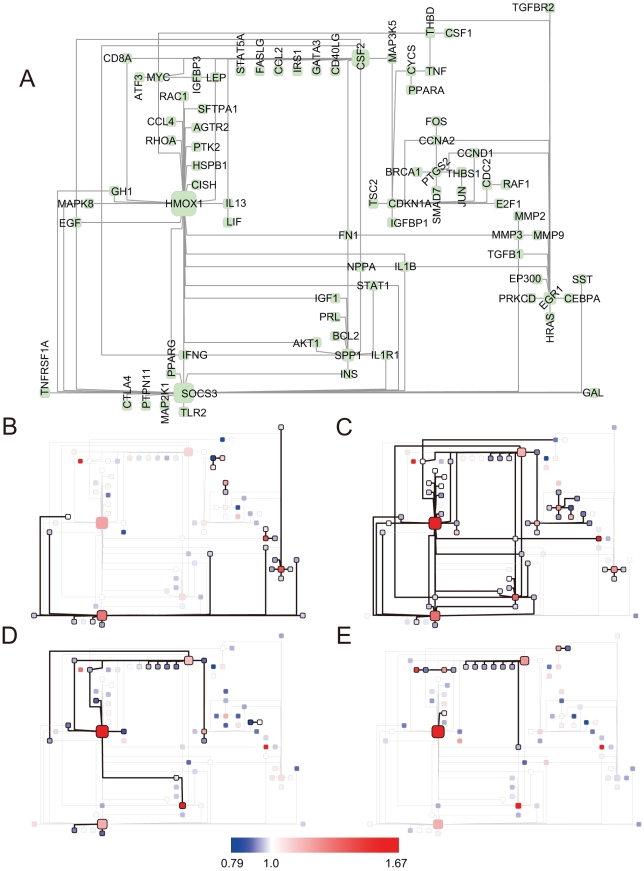
Changes in ON/OFF states of BTSs for time series data for human normal bronchial epithelial cells exposed to smoke. A: Toggling inferred from time-dependent data (ArrayExpress ID: E-GEOD-10700 and E-GEOD-10718) for the mRNA expression of normal human bronchial epithelial cells exposed to cigarette smoke for 24 hours. B: 2 hrs after exposure start, C: 4 hrs after exposure start, D: 8 hrs after exposure start, E: 24 hrs after exposure start. The nodes represent genes and the edges between them represent the pairing of bistable toggle switches. The colors of nodes were automatically assigned as a continuous color gradient from red for ON (upregulated) to blue for OFF (downregulated) according to relative gene-expression levels of the nodes. In Figs. 4B–E, the BTS pairs framed by thick lines are pairs with significant toggling scores at that time.

Figure 5A depicts the toggling of BTS ON/OFF states inferred from time-dependent data (ArrayExpress ID: E-GEOD-10700 and E-GEOD-10718) for the mRNA expression in normal human bronchial epithelial cells exposed to cigarette smoke for 24 hours. Toggling began at 2 hours (Fig. 5B) and was observed most frequently at 4 hours (Fig. 5C). SOCS3 (suppressor of cytokine signaling 3) was observed early, while BTSs related to HMOX1 (heme oxygenase 1), CSF2 (colony stimulating factor 2), and SPP1 (secreted phosphoprotein 1) were observed throughout the 24-h period.

SOCS3 inhibits cytokine signaling via the JAK(Janus kinase)/STAT(signal transducers and activators of transcription) pathway. Recent research has demonstrated that the activation of SOCS3 in the lung occurs during the acute inflammatory response [46]. Frequent hypermethylation in the CpG islands of the functional SOCS3 promoter has been found in lung-cancer tissue samples to correlate with its transcription silencing [47]. The OFF states of EGF (epidermal growth factor) and MAPK8 (mitogen-activated protein kinase 8) were linked to the ON states of CSF2 and HMOX1, which became the main players at four or more hours of exposure. CSF2 and HMOX1 were connected through several genes in the OFF state, including IL13 (interleukin 13), IFNG (interferon gamma), and FN1 (fibronectin 1), which are related to inflammatory responses and wound healing.

**Figure 6:**
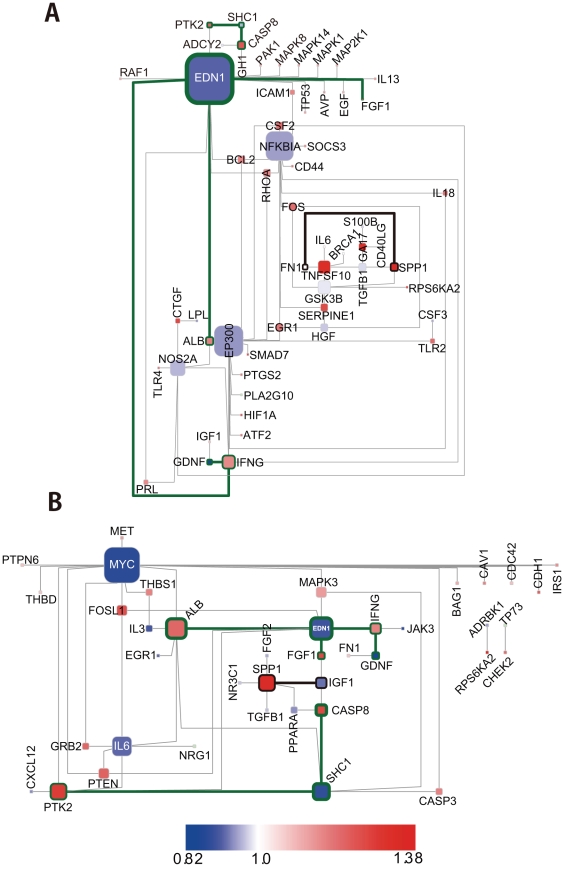
Changes in ON/OFF states of BTSs for lung cancer. The state of BTS toggling determined by comparing mRNA expression data (ArrayExpress ID: E-GEOD-10072) for normal lung tissue with that for lung-cancer patients with a history of smoking (former smokers) (Fig. 6A) and that for lung-cancer patients still smoking (current smokers) (Fig. 6B). The nodes and genes surrounded by bold black frames are those also shown in Fig. 5A. The nodes and edges surrounded by bold green frames are found in the former smokers as well as the current smokers. The nodes represent genes and the edges between them represent the pairing of bistable toggle switches. The colors of nodes were automatically assigned as a continuous color gradient from red for ON (upregulated) to blue for OFF (downregulated) according to relative gene-expression levels of the nodes.

Figure 6 illustrates the state of BTS toggling for a comparison of mRNA expression (ArrayExpress ID: E-GEOD-10072) in non-small cell lung carcinoma (NSCLC) patients with a history of smoking (Fig. 6A) along with those currently smoking (Fig. 6B) with mRNA expression seen in normal lung tissue. The bold black frames surround molecules that are also in the BTS molecules whose toggling is shown in Fig. 5A.

ON/OFF patterns of FN1-SPP1 (Fig. 6A) and IGF1-SPP1 (Fig. 6B) were observed in the data gathered in experiments exposing normal human bronchial epithelial cells to cigarette smoke. SPP1 is a secreted integrin-binding glycoprotein that is overexpressed in various tumors and has been reported to be involved in tumorigenesis and metastasis. High expression of SPP1 is a significantly unfavorable prognostic factor for the survival of patients with NSCLC [48].

In addition, although some EDN1(endothelin-1)-related BTS pairs and SHC1(Src homology 2 domain containing transforming protein)-related BTS pairs are shared in lung cancer tissue in current and former smokers, a considerable number of differing patterns are evident. This suggests that the mechanisms for carcinogenesis differ depending on the lengths of time that current and former smokers have smoked. EDN1, which is a hypoxia-inducible angiogenic growth factor for surrounding epithelial and endothelial cells, plays an important role in cancer-stromal interactions and tumor progression, and its expression is related to poor prognosis in NSCLC [49].

Small molecules that can put these BTS pairs into normal ON/OFF states might be useful in preventing the progression of lung cancer in both current and former smokers.

### Hepatocellular carcinoma

Hepatocellular carcinoma (HCC) is a primary cancer that originates in hepatocytes and typically follows cirrhosis or chronic-hepatitis virus infections [50], and the most significant risk factors for HCC are chronic infections with either hepatitis B virus or hepatitis C virus (HCV).

**Figure 7:**
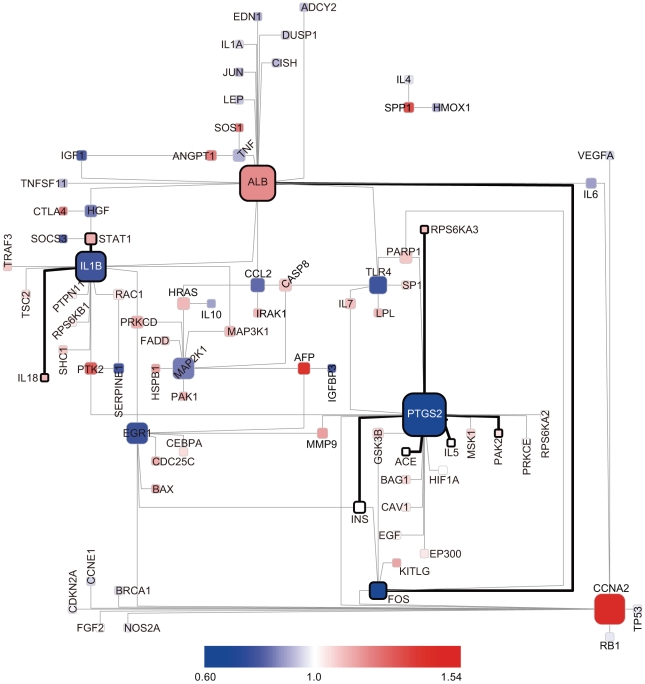
Changes in ON/OFF states of BTSs in dysplastic liver tissue and hepatocellular carcinoma. BTS toggling graph comparing the mRNA expression data (ArrayExpress ID: E-GEOD-6764) of normal liver tissue with that of precancerous and cancerous liver tissue. The nodes and edges surrounded by the bold lines are BTSs for which toggling was observed when comparing dysplastic liver tissue, a precursor of liver cancer, with normal liver tissue. The nodes represent genes and the edges between them represent the pairing of bistable toggle switches. The colors of nodes were automatically assigned as a continuous color gradient from red for ON (upregulated) to blue for OFF (downregulated) according to relative gene-expression levels of the nodes.

Figure 7 is a BTS toggling graph in which mRNA expression data (ArrayExpress ID: E-GEOD-6764, [51]) for tissues from patients with HCV-induced dysplasia and HCC are compared with mRNA expression data for normal liver tissue. The molecules surrounded by bold lines are BTSs for which toggling was observed when comparing dysplastic liver tissue (cirrhotic tissue and dysplastic nodules), a precursor of liver cancer, with normal liver tissue. The two tissue types share many BTSs associated with PTGS2 (prostaglandin-endoperoxide synthase 2; COX-2) and IL1B (interleukin 1, beta). It has been demonstrated that the expression pattern of PTGS2, a key enzyme of the prostaglandin metabolism, is closely correlated with the differentiation grade of HCC [52]. Nonsteroidal anti-inflammatory drugs targeting PTGS2 have been shown to inhibit the proliferation of cultured hepatocellular cancer cells by inducing cell-cycle arrest [53].

**Figure 8:**
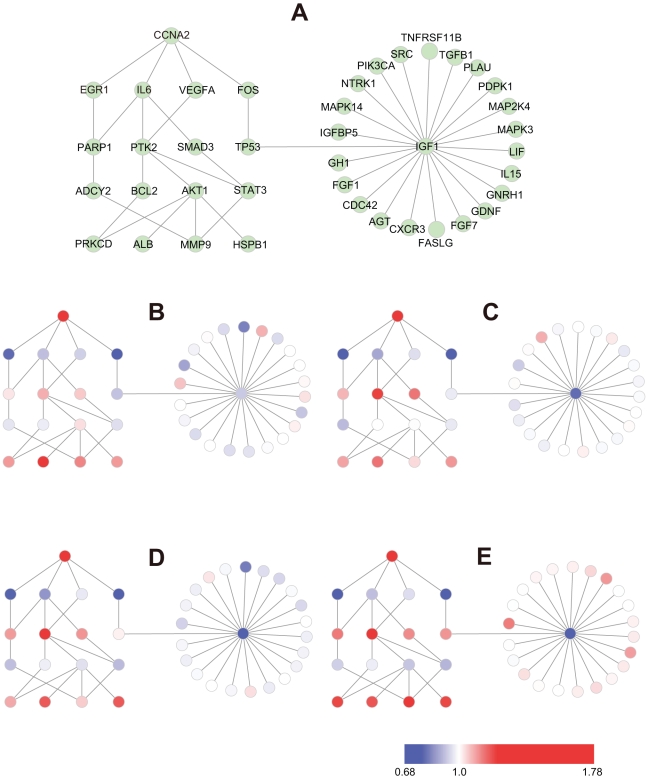
Rippling of toggling of CCNA2-related BTS during malignant transition of HCC. Fig. 8A: A network of CCNA2-related BTS pairs selected from the data used in Fig. 7A. Fig. 8B–E: The nodes represent genes and the edges between them represent the pairing of bistable toggle switches. The colors of nodes were automatically assigned as a continuous color gradient from red for ON (upregulated) to blue for OFF (downregulated) according to relative gene-expression levels of the nodes. B: very early HCC, C: early HCC, D: advanced HCC, E: very advanced HCC. Note that the ON/OFF status of TP53-IGF1 was changed in advanced HCC.

When HCC tissue was compared with healthy liver tissue, toggling was most evident for CCNA2(cyclin A2)–related BTSs (Fig. 7) We therefore analyzed how the toggling of CCNA2-related BTSs rippled out to other BTS pairs during the malignant transition of HCC (Fig. 8).

CCNA2 activates CDC2 or CDK2 kinases and regulates the cell cycle positively by promoting G1/S and G2/M transitions in both the G1 and G2 phases of the cell cycle [54], while EGR1 (early growth response gene 1) has suppresses transformation [55]. The upregulation of CCNA2 and downregulation of EGR1 might thus play a key role in the dysregulation of normal growth in HCC carcinogenesis [56]. The downregulation of IL6 (interleukin 6) is involved in dysregulation of the immune response in early carcinogenesis.

After the toggling of CCNA2-related BTSs but still in the early stage of carcinogenesis, the OFF state of IL6 is related to the ON states of PTK2 and SMAD3 (SMAD family member 3). PTK2 and SMAD3 play important roles in cell growth and the activation of intracellular signal transduction pathways, suggesting that cell proliferation might accelerate during this stage.

Toggling of PTK2(ON)-BCL2(OFF) was observed in advanced and very advanced stages. BCL2 (B-cell CLL/lymphoma 2) suppresses apoptosis, and the downregulation of BCL2 might be involved in the acceleration of apoptosis in cancer cells.

Notably, the ON/OFF state of the TP53-IGF1 BTS was changed from “OFF-OFF” to “ON(TP53)–OFF(IGF1)” in advanced HCC. And in very advanced HCC, almost all IGF1-related BTS pairs demonstrated “ON(other)–OFF(IGF1)” patterns.

In the very advanced stage, many IGF1(insulin-like growth factor-1)-related BTS pairs demonstrated significant ON/OFF changes. The liver is the main source of IGF1, and the development of HCC is accompanied by significantly reduced serum IGF1 levels [57]. The downregulation of IGF1 and upregulation of a set of another pair of genes might affect a wide variety of cellular functions.

## Discussion

We constructed bistable switch networks, compared their ON/OFF states with those of control (healthy) samples, and found that their states changed with disease progression and differed between patient subtypes. Since most disease states exhibit a certain level of resilience against therapeutic intervention, each can be considered to be homeostatic to some extent. This homeostasis implies the robust status of a dynamical network and could not be maintained without mechanisms that drive a network to maintain a certain state. One such mechanism is a bistable switch, so we should look for sets of bistable switch circuits in large-scale protein interaction networks.

Our analysis revealed that BTS states change with disease progression, and the implications of this are far reaching. For example, it might be possible to prevent or delay disease progression by perturbing one or more such switches. Such switches may be novel drug-target candidates for controlling disease progression. Analysis of the ON/OFF states of genes constituting bistable circuits revealed similarities between disease subtypes.

While our analysis has provided insightful information, it has shortcomings. First, the network topologies were based on commercial databases created using a text-mining system. This means that the details of the molecular interactions were not verified. The development of a more accurate interaction database would enable more precise and accurate analysis of bistable network behaviors and of the contributions of switch circuits to those behaviors. Second, the analysis was based solely on network topologies—no parametric features were considered. Although topological analysis enabled us to identify circuits exhibiting bistable behavior, whether circuits exhibiting bistable behavior apparently exhibit bistable behavior depends on the kinetic parameters associated with each interaction [58].

Using microarray data, we determined that the pairs of genes in the circuits we identified are polarized into ON and OFF states. Two mutually inhibitory nodes polarized into ON and OFF states do not function as a bistable switch if both genes are ON or OFF. This is why we focused on BTSs, which demonstrated “ON/OFF” or “OFF/ON” states. We should, however, note that the “ON/ON” states of some BTSs play important roles in mammalian embryogenesis [59], T-cell differentiation [60], and visual-system specification [61].

Cluster analysis of transcriptome data in microarrays is useful for classifying disease characteristics according to differences in expression patterns. Although several disease types that are difficult to classify morphologically have been classified using this approach, the rules underlying the cluster structure of the data are unclear, and the importance of each of the molecules in a cluster cannot be determined with a reasonable degree of certainty. The analysis of changes in gene-expression levels can also be used to create a list of molecules whose levels increase or decrease significantly over time or whose levels differ significantly between healthy and diseased tissues. Although examinations of gene interrelations using gene-ontology classification and analysis of the classification results using network diagrams have led to a greater degree of understanding of the changes in molecular networks, it is difficult to infer the meanings of biological interactions between molecules.

Our proposed method (i.e., focusing on BTS ON/OFF changes) takes as the starting point the interactions between molecules. This makes it easy to infer biological meaning and makes it possible to analyze time-dependent data for time periods corresponding to that of disease progression (from hours to years). In addition, while conventional methods sometimes neglect molecules that are downregulated, our method places equal importance on both increases and decreases in expression.

DNA microarray technology makes it possible to study the expression of thousands of genes at the same time, but much of the microarray data consists of low signal intensities that can produce erroneous gene expression ratios between control and experimental samples [62]. The distribution of the ratio of two random variables approaches a Cauchy, or Lorentzian, distribution, which has longer tails than Gaussian distributions [63], [64]. In our results, far more BTS pairs had significant toggling scores than did random gene pairs, but a considerable number of random gene pairs did show significant ON/OFF changes. We should therefore consider the possibility of random error in the analysis of BTS pairs.

We used the transcriptome of normal tissue as the control in our analyses. This means that the identification of the molecular ON/OFF states inherent to normal tissue was unclear. Even if the ON/OFF state of a molecular pair for a certain switch is important for a particular tissue, if this state is retained in the diseased tissue, we would be unable to detect it in the present study because the ON and OFF states are not mutually exclusive. Therefore, molecules exhibiting even the slightest change are emphasized while those showing no change are ignored. We aim to overcome this drawback by identifying what types of ON/OFF changes occur in switches when embryonic stem (ES) cells or iPS cells undergo differentiation.

Since proteins are responsible for cell function, the ON/OFF state of a molecule must be determined at the protein level when searching for molecular-network structures mediating cell functions. Because there are more than 20 control steps along the way from mRNA to functional proteins [65], the reported expression levels of mRNA do not always agree with those of proteins—their translated products [66]. And even if there were a quantitative correlation between the levels of mRNA and functional protein, the efficiency of the translation process would be greatly affected by factors such as structural change and protein localization. Proteomics data for proteins in different cellular contexts is useful but is available for only some proteins. Transcriptome data analysis is the only method currently available for examining molecular networks on a large scale, but when testing the quality of BTS pairs in the future we will use all the relevant available data for the target proteins. Furthermore, to ensure bistability, the hysteresis phenomena must be confirmed when a perturbation has vanished. By conducting time-scale experiments in both directions when applying and removing perturbations, we should be able to further test the quality of BTS pairs.

Despite its shortcomings, the approach presented here provides useful insights into the states of biological networks, insights that may lead to discovery of novel drug targets and therapeutic interventions.

## Materials and Methods

### Preparation of basic interaction datasets

The lists of molecular interactions were constructed using the Ariadne Genomics ResNet human protein interaction database (ver. 3.0) compiled, using MedScan [67] natural language processing technology, from more than 13,000,000 PubMed abstracts and 43 publicly available full-text journals. The database contains data on over 200,000 objects (proteins and small molecules) and over 100,000 interactions.

The interactions can be divided into two major classes: direct physical interactions (binding, protein modifications, and promoter binding) and indirect regulatory interactions (regulation, expression regulation, direct regulation, molecular transport regulation, and molecular synthesis regulation). MedScan also extracted information on the relation direction and the effect on the target molecule. The “Effect” attribute has three possible values: “positive,” “negative,” and “unknown.” The BTS pairs were extracted from the database on the basis of five rules.

1. Nodes are limited to genes and proteins only.
2. Edges are limited to “Regulation,” “Expression,” and “DirectRegulation.”
3. “Unknown” edges in the “Effect” attribute are omitted.
4. Edges extracted from fewer than three references are omitted.
5. If there is a positive and negative attribute in the same direction, the edge is extracted from additional references.

We extracted 19,178 relationships involving 3,682 genes (basic interaction datasets).

### Extraction of candidate bistable toggle switches

Using basic interaction datasets, we extracted possible network motifs for toggle switches. We defined these motifs as follows.

The type-1 BTS contains two genes that have positive autoregulation and inhibit each other's expression. The type-2 BTS also contains two genes that suppress each other's expression, but each gene also has a positive or negative loop with the other gene. One of the four subtypes of type-2 BTSs (corresponding to the four possible combinations of double positive and/or negative feedback) shows the same function as the type-1 BTS. The type-3 BTS was based on a theoretical study of the modeling of genetic switches with positive feedback loops [37]. The BTS motifs are illustrated in Fig. 2, and we extracted 6585 BTSs (see supporting Table 1).

### Analysis of toggling

We used mRNA microarray data to examine the changes in the ON/OFF states of BTS candidates. CEL format files or tab-limited text files were downloaded via ArrayExpress (http://www.ebi.ac.uk/arrayexpress/), which is a public repository provided by the European Bioinformatics Institute [68]. We only used microarray data obtained from experiments with humans and with platforms of Affymetrix HG-U133A&B (631 sets) and HG-U133Plus2.0 (404 sets). These data were normalized and summarized using the robust multichip analysis method [69] implemented in the Affymetrix Expression Console software.

The toggling of a BTS pair was defined as instances in which the mRNA levels of a sample increased for one molecule of the pair and decreased for the other. To remove background noise, we calculated the toggling score using
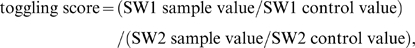



where SW1 and SW2 are the two molecules in alphabetical order. Changes in the ON/OFF states were considered significant when the toggling score was more than two standard deviations greater than the mean of all the toggling scores.

### Network visualization

For pathway visualization, we used Cytoscape (Version 2.6.3), which is widely used open-source software for visualization and analysis of networks [70]. The nodes in the visualized BTS network represent genes, the edges between nodes represent the pairing of bistable toggle switches, and the color of nodes were automatically assigned as a continuous color gradient from red for ON (upregulated) to blue for OFF (downregulated) according to relative gene-expression levels of the nodes.

## Supporting Information

Protocol S1 List of BTS pairs SW1 and SW2 are the two molecules comprising a BTS pair in alphabetical order.
(0.16 MB XLS)

Protocol S2 Cytoscape session file for Figure 3.
(0.09 MB ZIP)
